# The Role of Anharmonicity in the HC*D Chromophore in Vibrational Circular Dichroism Spectra and Optical Rotation Data

**DOI:** 10.1021/acs.jpca.5c03064

**Published:** 2025-07-14

**Authors:** Marco Fusè, Giovanna Longhi, Giuseppe Mazzeo, Julien Bloino, Sergio Abbate

**Affiliations:** † Dipartimento di Medicina Molecolare e Traslazionale, Università di Brescia, Viale Europa 11, Brescia 25123, Italy; ‡ Scuola Normale Superiore, Piazza dei Cavalieri, Pisa 56125, Italy; § Research Unit of Brescia, Istituto Nazionale di Ottica (INO), CNR, c/o CSMT, VIA Branze 45, Brescia 25123, Italy

## Abstract

The vibrational circular dichroism (VCD) spectra in the CD-stretching region of (*R*)-(−)-neopentyl-1d-halides (Cl and Br), (*R*)-(+)-1-exo-d_1_-camphor, and (*S*)-(+)-1-phenylethane-1-d_1_ (and (*S*)-(+)-1-phenylethane-1,2,2,2-d_4_) are correctly interpreted by properly including anharmonicity, both mechanical and electrical, with DFT calculations. This result is noteworthy, since the spectroscopic range containing the CD-stretching transitions is always rich in features attributed to overtone and combination transitions. The importance of the reported simulations is thought to go beyond the cases discussed here and to be applicable to other molecules containing the HC*D fragment and, more generally, CD, a point that is rising in importance after the FDA’s approval of deuterated drugs. Furthermore, proper treatment of anharmonicity helps in the interpretation of other observables, like e.g., specific optical rotations (OR).

## Graphical abstract

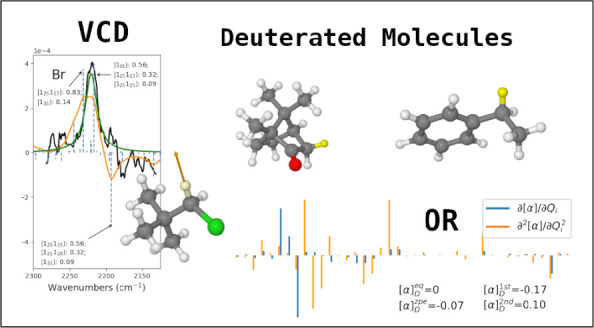

## Introduction

The addition and use of deuterium in drugs, with the aim of obtaining a slightly different behavior from hydrogen, much in the same way as previously done with fluorine, is actively and successfully pursued^1–5^ in chiral drugs, starting from the first one approved by the FDA, which is useful in treating chorea associated with Huntington’s disease.^6^ This requires physical methods, including spectroscopic ones, to monitor its presence and to help understand why deuterium may be important in chiral molecules. This need was already evident when Mosher contacted researchers in the nascent field of vibrational circular dichroism (VCD) spectroscopy to study chiral neopentyl-1d-halides, of the structure *t*-butyl-C*HD-X (X= Cl, Br, etc.), whose optical rotation (OR) showed very little differences.^7,8^ Those studies were the continuation of an interesting story, stimulated by the ideas of Kirkwood and initiated by Kirkwood himself,^9,10^ followed by Eliel,^11^ Alexander and Pinkus,^12^ and Fickett,^13^ combining experiment and computations to understand the OR data of deuterated chiral molecules defined by the general formula R_1_R_2_C*HD. It is indeed worth mentioning that Fickett^13^ had pointed out the special role of C*H- and C*D-stretching vibrational modes in the observed and calculated tiny values for OR on top of polarizability differences of R_1_, R_2_, H, and D groups, as proposed by Kirkwood (vide infra).

The first such system, studied by means of VCD in the C*D-stretching region, was (*R*)-(−)-neopentyl-1d-chloride.^14^ The result was a structured VCD band with an overall rotational strength of the expected order of magnitude. Its understanding spurred the development of the first computational and heuristic models of VCD, including the fixed partial charge (FPC) model,^15^ the localized molecular orbital (LMO) model,^16^ and the charge flow model.^17,18^ The VCD measurement of the second member in the series (X = Br) gave, somewhat unexpectedly, a VCD band of opposite sign in the CD-stretching region that could not be explained by the existing models.^18,19^

In a recent paper,^20^ we saw that combination bands and overtones are present in large numbers in the 1700–2400 cm^−1^ range, thus encompassing the CD-stretching region. Incidentally, in the early stages of the development of VCD, Stephens and Clark had already called attention to the possibility that anharmonicity could play an important role due to the presence of overtone and combination vibrational transitions in the same spectroscopic region as the CD-stretching, between 1900 and 2400 cm^−1^, as they had observed for camphor and a deuterated isotopologue, as well as in a couple of other cases containing the HC*D moiety.^21^

Building upon our experience on anharmonicity in VCD, both in the mid-IR region, in the CH/OH stretching regions, including high overtones, and in the near-infrared (NIR) and visible spectrum,^20,22–24^ we propose here a systematic study of the effects of anharmonicity on a series of deuterated compounds, starting from the neopentyl-1d-halides, to see if they could explain the differences in signs between Cl and Br, including an analysis of the effects of deuteration in the VCD spectrum of camphor, following the steps of Stephens and Clark. As part of this study, we have also considered a third example, closely related to the case of neopentyl-1d-halides, (*S*)-(+)-1-phenylethane-1-d_1_, and (*S*)-(+)-1-phenylethane-1,2,2,2-d_4_, for which the VCD spectra had already been recorded in that region as part of a full VCD-IR-Raman study of a series of four deuterated phenylethanes.^25,26^

In this work, we thus report and discuss the computational anharmonic results for (*R*)-(−)-neopentyl-1d-halides, (*R*)-(+)-1-exo-d_1_-camphor, and (*S*)-(+)-1-phenylethane-1-d_1_, (*S*)-(+)-1-phenylethane-1,2,2,2-d_4_ (depicted in Figure 1), where we have made use of the most recent updates on vibrational perturbation theory at the second order (VPT2) and its generalized (GVPT2) variant,^20,24,27,28^ as implemented in Gaussian 16,^29^ which makes use of the MFP (magnetic field perturbation) theory developed by Stephens.^30^ In an effort to connect to the early works on these systems, we will discuss whether it is possible to talk about a VCD HC*D inherently symmetric chromophore, in a parallel way as had been defined by Moscowitz^31^ for ECD, as opposed to inherently dissymmetric chromophores (in the latter case, a series of examples of excitonic-like vibrations were proposed for the VCD^32^), and whether the VCD harmonic and anharmonic results can shed some light on OR data.

**Figure 1:**
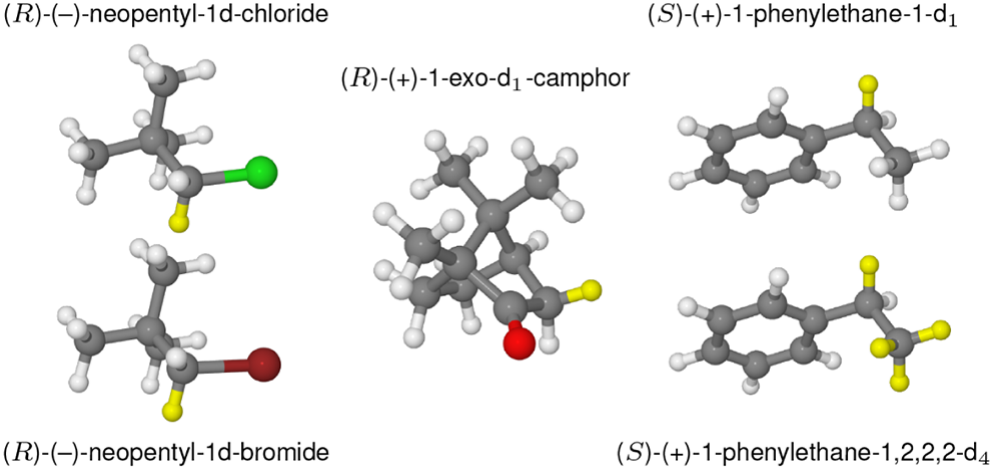
Graphical representations of the investigated molecular systems. The deuterium atoms are colored in yellow.

At the end of this introductory note, we shall mention that VCD spectroscopy has recently been applied to deuterium-labeled compounds by other groups^33–36^ confirming the growing interest in this field.

## Experimental and Computational Methods

All experimental data, except for two VCD and IR spectra, were taken from the literature^19,21,26^ and digitally processed, employing, when needed, further information from PhD theses,^18,37^ on which some papers were based. We only wish to report that the VCD data of (*R*)-(−)-neopentyl-1d-halides were taken for neat liquid samples, while those of (*R*)-(+)-1-exo-d_1_-camphor and deuterated (*S*)-(+)-1-phenylethanes were taken in CCl_4_ solutions. The experimental VCD and IR spectra for (*R*)-camphor and (*S*)-camphor, undeuterated species, have been obtained for this work in Brescia’s lab with an FTIR-VCD Jasco FVS 6000 instrument (6000 accumulations for each species and solvent subtraction, with solvent spectra run under the same conditions). The VCD and IR data for (*R*)-camphor have been found to be quite similar to those of Stephens and Clark.^21^

Electronic structure calculations, including frequency-dependent optical rotations at the sodium D-line (589 nm), were performed at the DFT level using the B3LYP^38,39^ functional in conjunction with def2-TZVP^40^ basis sets, utilizing the Gaussian 16 suite of quantum chemical programs.^29^ For the camphor molecule, to improve the agreement with the experimental results, a hybrid scheme was employed: harmonic frequencies were computed at the B2PLYP^41^/def2-TZVP level of theory.^42^ Anharmonic calculations were performed at the pure VPT2 level for vibrational averages^43,44^ and using the GVPT2 scheme for the IR and VCD spectra. The contributions of hindered rotors, which are poorly described by the polynomial expansions within the Cartesian-based normal-mode picture used in VPT2,^45^ were removed from all anharmonic calculations and from the calculation of the vibrational correction to the optical rotation.^46^ In IR and VCD anharmonic calculations, resonances were automatically identified as per the protocol detailed in ref 47 and implemented in the development version of Gaussian. In this framework, the resonant terms were then removed and reintroduced through a variational step.^44,48^ The latter step was crucial to reproduce the resonant interactions of the C*D-stretching fundamental with close by overtones and combinations. Lorentzian broadening functions with half-widths at half-maximum values of 7 and 10 were used to simulate the band shapes in the CD and CH stretching regions, respectively.

## Results and Discussion

### HC*D in (*S*)-(−)-Neopentyl-1d-Halides

In Figure 2, we report the superposition of experimental and computational (harmonic and anharmonic) IR and VCD spectra in the CD-stretching region for (*R*)-(−)-neopentyl-1d-chloride and (*R*)-(−)-neopentyl-1d-bromide. One may see that the harmonic, scaled-frequency DFT-calculated spectra predict the same sign for the VCD spectra of the two molecules, at odds with experimental data. Incidentally, very similar VCD spectra are predicted in the mid-IR and CH-stretching regions for the two molecules (see Figure S1), with little differences. In the absence of experimental data for these regions and since they are not directly relevant to the present study, they will not be further investigated. On the contrary, the anharmonic GVPT2 calculations not only allow one to predict the correct overall sign for the spectrum in the CD-stretching region, but they also predict the overall spectral shape in general agreement with the observed one, evidencing possible features overlooked in the original work.^18,19^ While we cannot exclude the possibility that some minor features arise from experimental artifacts, we tentatively attribute meaning to these minor features in terms of two-quanta combination bands. It is interesting to note that the three most intense calculated VCD transitions have the same pattern in the two molecules: the two transitions above 2200 cm^–1^ being positive and the one close to (for (*R*)-neopentyl-1d-chloride) or slightly below 2200 cm^–1^ (for (R)-neopentyl-1d-bromide) being negative. Small differences in intensity and frequency are observed in the two cases, caused by minor anharmonic interactions between the fundamental CD-stretching state 1_35_ and two-quanta combination states (mainly 1_25_1_15_ or 1_25_1_17_, vide infra), explaining the sign change between the two halide molecules. Remarkably and unlike the other two following cases, the VCD sign of the interacting two-quanta transitions is crucial in determining the intensity of the major signal observed in the experiments among the three main components of the VCD spectra in that region (see Table S1 for the rotational strength values of the deperturbed transitions). These three lines are also the main contributors to the IR absorption spectra, which are very well predicted in the total intensity and shape, the latter being narrow but asymmetric due to evident shoulders. We are confident that further studies on these and other deuterated molecules with higher signal-to-noise ratio (S/N) spectra will deepen the understanding of their anharmonic vibrational features.

**Figure 2:**
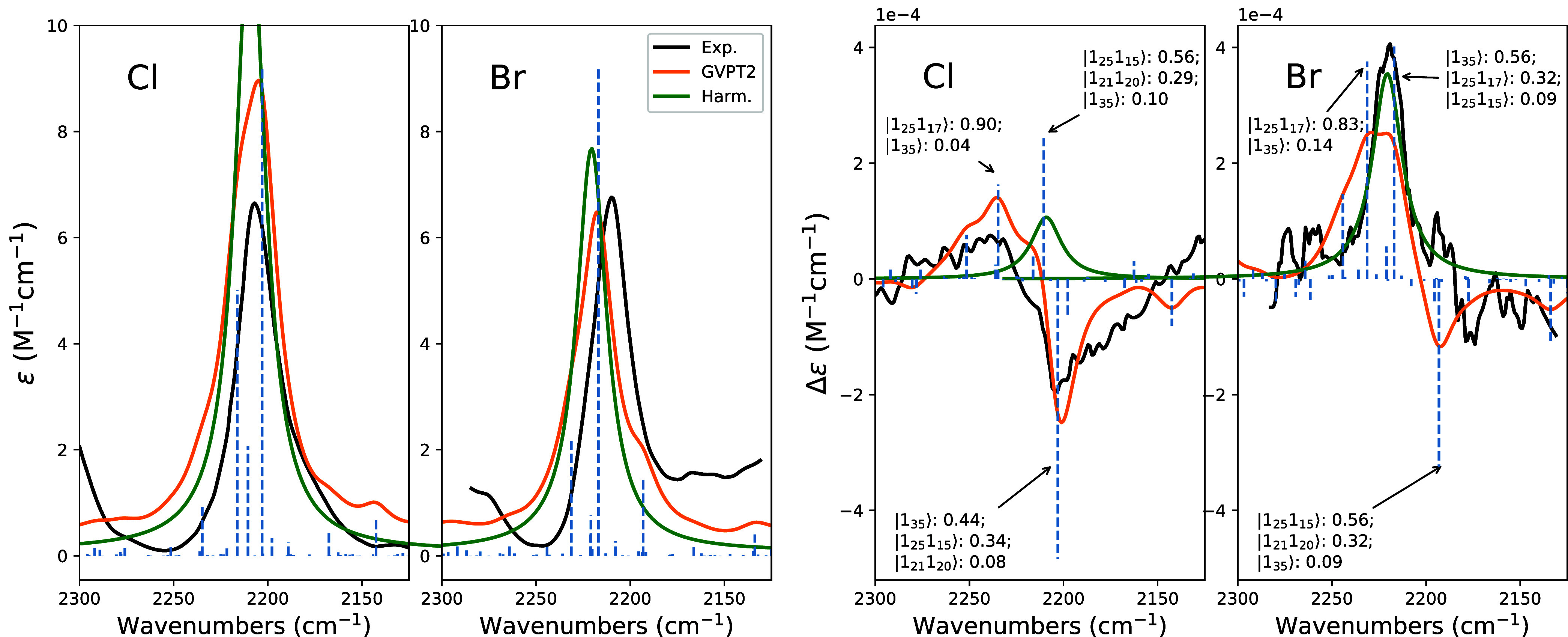
Comparison of experimental and calculated IR (left) and VCD (right) spectra of (*R*)-(−)-neopentyl-1d-chloride and (*R*)-(−)-neopentyl-1d-bromide in the CD-stretching region. Experimental spectra are in black, calculated harmonic spectra are in green and calculated anharmonic spectra are in orange. For the latter, we also report the stick spectra for the 20 most relevant transitions and the assignment of the three most intense lines. Dirac’s notation for normal mode eigenvectors is employed with big numbers indicating the number of vibrational quanta and subscripts indicating the vibrational normal mode, null quanta being ignored, while the decimal number represents the squared coefficient in the variational state. Calculated harmonic spectra were scaled by a 0.95 factor, to bring them in the vicinity of the experimental band. Experimental data adapted from refs 18, and 19.

### HC*D in (*R*)-(+)-1-exo-d_1_-camphor

In Figure 3, we report the results for the second investigated system. Due to the evidently large number of experimental features in a region where only one harmonic transition is expected (which motivated Stephens and Clark to run spectra also for undeuterated (*R*)-camphor), we did not find it instructive to report the results for the harmonic calculations (see Figure S2). The large number of combination transitions in this spectroscopic region (see both the left and right panels of Figure 3), each one of a nonnegligible intensity, leads to multiple possible interactions (especially of the Fermi type) with the fundamental CD-stretching mode transition. A graphical representation of all the active Fermi resonances identified, removed, and then reintroduced in the variational step is reported in Figure S3.

**Figure 3:**
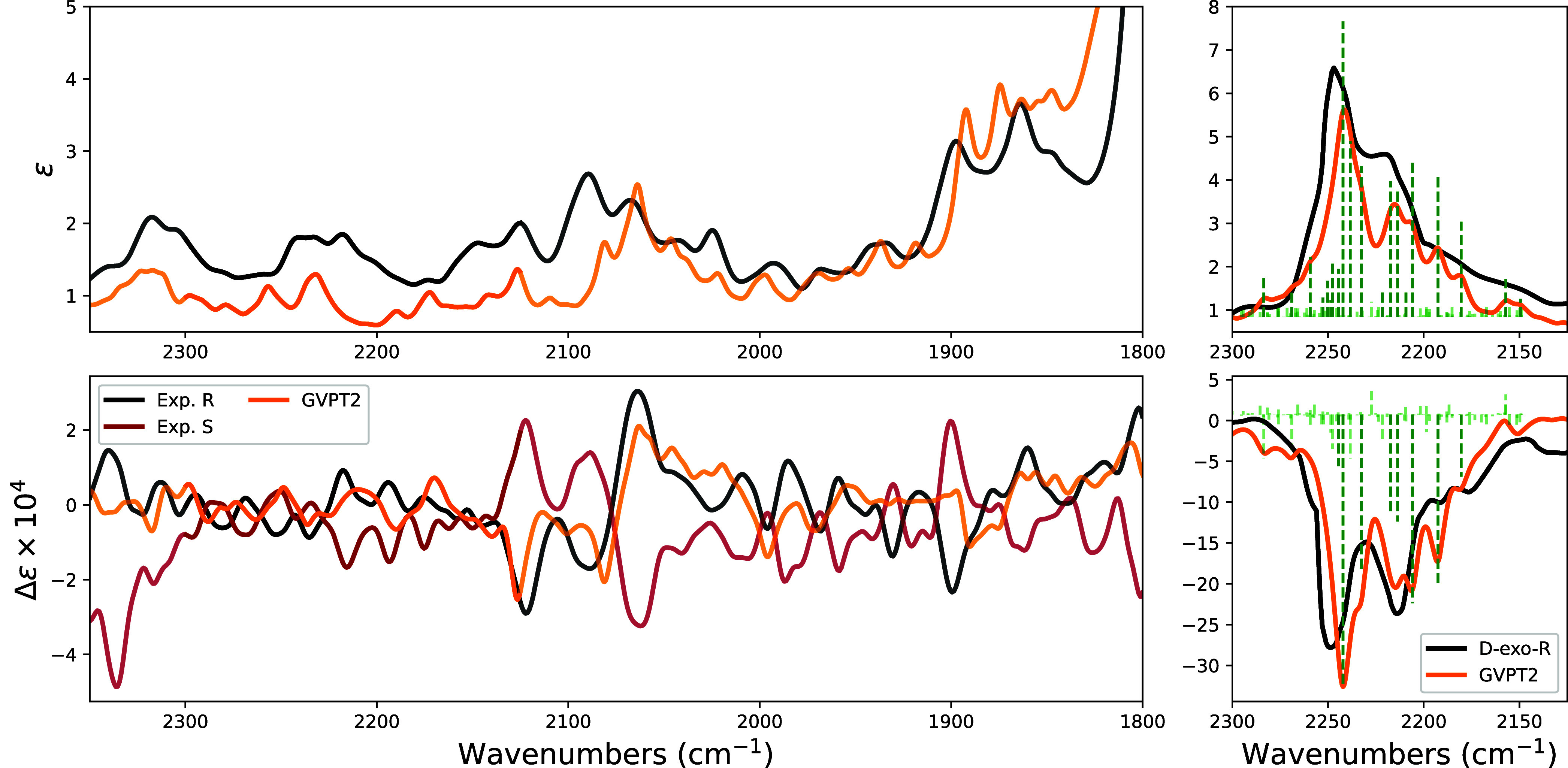
Comparison of experimental and calculated IR and VCD spectra of (*R*)-(+)-camphor and (*S*)-(−)-camphor (left) and of (*R*)-(+)-1-exo-d_1_-camphor (right) in the 2300–1900 region. Experimental spectra are in black for (*R*) and red for (*S*), calculated anharmonic spectra are in orange. For the latter, we also report the stick spectra for the 20 most relevant transitions. Experimental data on the right are redrawn from ref 21, while those on the left of the figure are original of this work (see the Experimental and Computational Methods section).

The bottom-left panel of Figure 3 (no deuteration) shows that VCD features due to combinations are in fact observed and well-predicted by our anharmonic treatment. On the right, in the region limited to the fundamental of the CD stretching mode, at least eight calculated transitions have large VCD and IR strengths, and the four most prominent observed VCD and IR bands/shoulders are quite well-predicted (the eight VCD transitions are highlighted in Figure S6 and described in Table S2). Unlike (*R*)-(−)-neopentyl-1d-halides, the VCD intensity of the fundamental transition is so dominant that the intensity redistribution through the Fermi resonances results in all nonnegligible transitions to have the same negative sign (see Figure S2for a comparison of the intensity before and after redistribution). Through the variational coupling (see Figure S4), the band is spread over a wider energy range and the intensity distribution explains the shape observed experimentally.

### HC*D in Deuterated Phenylethanes

The case of deuterated (*S*)-(+)-1-phenylethanes appears intermediate between the two cases presented above, (*R*)-(−)-neopentyl-1d-halides and (*R*)-(+)-1-exo-d_1_-camphor. Indeed, not only the experimental VCD CD-stretching region appears monosignated as with (*R*)-(+)-1-exo-d_1_-camphor, but the positive feature is also weak, and overall, the negative sign is seen to prevail. Together with the many combination features present, these characteristics makes the phenylethane case more akin to the camphor case (Figure 4). This is particularly true in comparison with (*R*)-(−)-neopentyl-1d-halides, with the overall negative sign prevailing in the CD-stretching region not only for (*S*)-(+)-1-phenylethane-1-d_1_ but also for other partially deuterated phenylethanes treated in ref 26. It is interesting that the aliphatic CH-stretching band, which is isolated and alone for (*S*)-(+)-1-phenylethane-1,2,2,2-d_4_ without overtone or combination features nearby, is positive; this looks reasonable, since the CH and CD bonds in the HC*D group are, broadly speaking, in a sort of enantiomeric relationship. The negative features in the CD-stretching region originate mainly from the Fermi interaction of fundamental transition 1_39_, which has a negative rotational strength before variational correction (see Figure S5), with a few combination transitions, among which one can single out 1_28_1_15_. The Fermi resonance pattern had already been recognized in the original publication of Havel et al. on deuterated phenylethanes^26,37^ and is confirmed here by full anharmonic calculations. As a final remark, we considered only one conformer, with the phenyl ring perpendicular to the CHD plane here, since no other conformers are appreciably populated, differently from what was originally assumed (shown in the bottom part of Figure S7).

**Figure 4:**
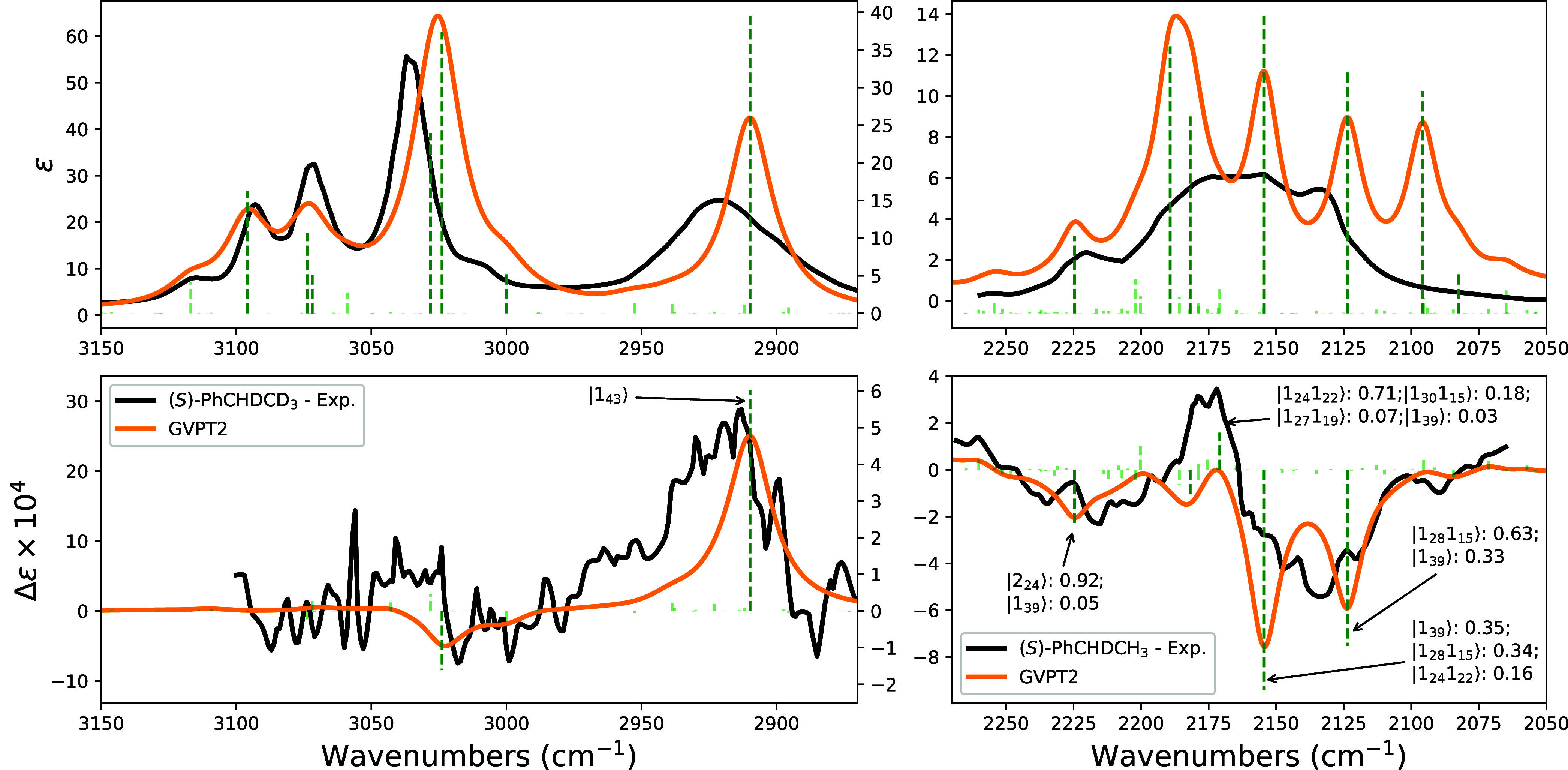
Comparison of experimental and calculated IR (top) and VCD (bottom) spectra of (*S*)-(+)-1-phenylethane-1,2,2,2-d_4_ in the CH-stretching region (left) and of (*S*)-(+)-1-phenylethane-1-d_1_ in the CD-stretching region (right). Experimental spectra are in black, calculated anharmonic spectra are in orange. In the last case we also report the stick spectra for the 20 most relevant transitions and the assignment of the four most intense lines. See the caption of Figure 2 for details on the notation.

To further appreciate the similarities and differences between the first and third cases presented above, we report in Figure S7 the graphical representations of the normal modes for the fundamental CD stretching and those involved in the relevant combination transitions in the cases of (*R*)-(−)-neopentyl-1d-chloride and (*S*)-(+)-1-phenylethane-1-d_1_. In the neopentyl case, it is clear that the two combination modes interacting with the CD-stretching fundamental (mode 35) involve one quantum in the bending mode of the CH bond in the HCD plane (mode 25) and one quantum in the out-of-HCD-plane twisting mode around the bisecting HCD line (mode 15). On a side note, the importance of these two modes, in the fundamental state, had already been pointed out in refs 18 and 19. A similar pattern is observed in the (*S*)-(+)-1-phenylethane-1-d_1_ case between the fundamental of mode 39 and overtone/combination transitions involving the vibrational “recoil” of the light H atom, with also the participation of antisymmetric CH_3_-bending modes. The consideration of these modes could be important for the assessment of the observed OR value.

For the sake of completeness and to show how anharmonic calculations account well for the observed spectra, including those with multiple CD (CH) groups, we report in Figure S8 the spectra of (*S*)-(+)-1-phenylethane-1-d_1_ in the CH stretching region and (*S*)-(+)-1-phenylethane-1,2,2,2-d_4_ in the CD stretching region.

In an effort to rationalize the VCD data, one could be tempted to consider that the effect of anharmonicity lies primarily in the broadening of the bands, with the emergence of new, low-intensity nonfundamental bands, while the sign is dictated by the configuration of the C* stereogenic carbon. This is not the case. In fact, for (*R*)-(−)-neopentyl-1d-chloride and for (*R*)-(+)-1-exo-d_1_-camphor, which is locally (*R*) for carbon 3, one has an overall (−) sign for C*D-stretching, while for (*R*)-(−)-neopentyl-1d-bromide and for (*R*)-(−)-1-phenylethane-1-d_1_ – even though we actually worked with the enantiomer (*S*)-(+)-1-phenylethane-1-d_1_ – one observes an overall (+) sign. The overall rotational strength appears to be related to more subtle effects, rooted in interactions, mostly anharmonic, with nearby groups.

### The Role of HC*D in Optical Rotation

This suggests that anharmonic effects may also be quite important in other chiroptical spectroscopies when the HCD group is the only chromophore. Considering a property like optical rotation, often used to assign the absolute configuration of the C* stereogenic carbon, we expect that precise calculations of the vibrational contributions should be crucial for its correct prediction, especially from terms originating from the C*D and C*H stretching transitions. As a matter of fact, in the cases of deuterated neopentyl halides and phenylethane, the observable optical rotation is necessarily connected to vibrations. This idea had been considered and discussed in the early work of Fickett,^13^ who adopted the model of interacting polarizable groups by Kirkwood^9,10^ and adapted it to R_1_R_2_C*HD molecules (see Section S2). The importance of polarizability was also noted in a previous work by one of us on (*S*)-(+)-1-phenylethane-1-d_1_ and other deuterated species.^26^ For this reason, we report in Table S3 the experimental polarizability values of some groups relevant to the molecules investigated here.

Nowadays, calculations of OR values at the DFT level have become a routine task, including vibrational corrections.^46,49–52^ Regarding the latter, we should simply note here that the vibrational averages at the VPT2 level have two components, one related to the expansion of the potential energy surface, called mechanical anharmonicity, and one dependent on the second derivatives of the property of interest, here, the electric dipole–magnetic dipole tensor, which will be simply referred to in the following as the electrical anharmonicity. More details are provided in Section S3. In Figure 5, the contributions to the vibrational corrections of each normal mode for (*R*)-(−)-1-phenylethane-d1 and for (*R*)-(−)-neopentyl-1d-bromide are reported (the other two cases are reported in Figure S9). Contributions from the mechanical anharmonicity [α]_D_^mec^ are limited, and the only relevant terms are associated with C*H and C*D vibrations, both bond-stretching or in-plane and out-of-plane HC*D-bendings. The situation is less obvious for the influence of the property surface curvature; several normal modes give nonnegligible contributions through the “electrical” term, influencing the sign and magnitude of $[\alpha {]}_{D}^{\mathrm{ele}}$ term.

**Figure 5:**
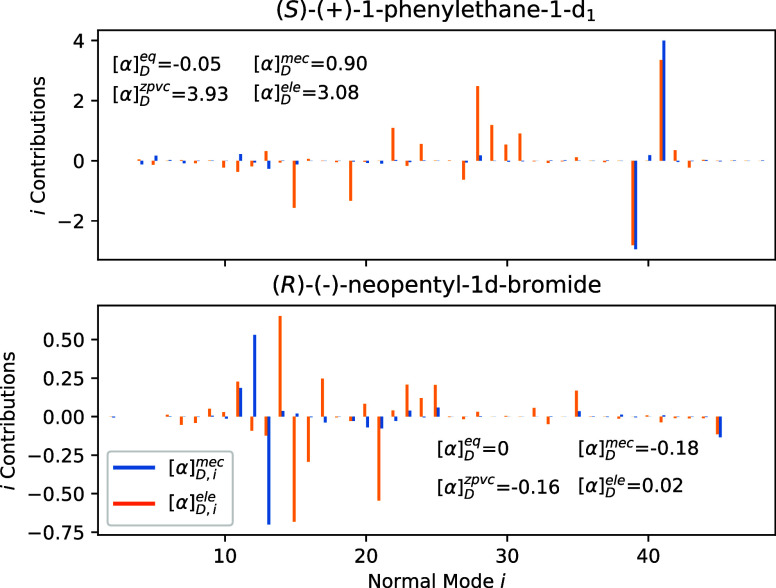
Graphical representation of the mechanical $[\alpha {]}_{D}^{\mathrm{mec}}$ and electrical $[\alpha {]}_{D}^{\mathrm{ele}}$ anharmonic contribution to the zero point vibrational corrected optical rotation calculated values for (*R*)-(−)-neopentyl-1d-bromide (top) and (*R*)-(−)-1-phenylethane-1-d_1_ (bottom) for each normal mode *i*. See Section S3 for the definition of the two terms.

The computed optical rotations, including temperature dependence,^45^ for the four molecules investigated here are compared to the experimental values in Table 1. Despite being simulated in the gas phase, these values are in good agreement with the experimental ones, reproducing the sign and general magnitudes of the observed signals. The least satisfactory agreement is obtained for the phenylethane molecules, for which the calculated values are overestimated. Besides environmental effects, which may play a role,^52,55^ the reason for this discrepancy is likely due to the lack of a proper description of the phenyl hindered rotor, which would have required an explicit averaging over the hindered rotation and a specific treatment of its coupling with the rest of the system.^45^

**Table 1:** Specific Optical Rotation Values Measured and Calculated at the Sodium D-Line (589 nm), [α]_*D*_ for the Molecules Under Study.^a^

(*R*)-neopentyl-1d-Cl^b^			(*R*)-3–1*d*_1_-exocamphor			(*S*)-1-phenylethane-1-d_1_ ^f^		
Exp.	Calc.		Exp.	Calc.		Exp.	Calc.	
	eq	Vibr.		eq	Vibr.		eq^h^	Vibr.
–0.25	0.00	–0.07	(+)^g^	+43.6	+46.4	+0.81	–0.05	+3.35

(*R*)-neopentyl-1d-Br^c^			(*R*)-camphor^d^			(*S*)-1-phenylethane-1,2,2,2-d_4_ ^f^		
Exp.	Calc.		Exp.	Calc.		Exp.	Calc.	
	eq	Vibr.		eq	Vibr.		eq^h^	Vibr.
–0.124	0.00	–0.15	+44.1	+43.6	+43.6	+0.79	–0.05	+3.30
			+59^e^					

			(*S*)-camphor^d^					
			Exp.					
			–43					

a All [α]­D are measured at 25 °C, except for phenylethanes, where they were measured at 20 °C. Experimental values are compared with equilibrium simulated values (eq.) and with the vibrational corrected one (Vibr.)^43,54^ Vibrational corrected OR values were computed accounting for thermal effect (298 K) and the terms related to the poorly described low-energy vibrations were removed.^46^ Data taken from:

b Stephenson et al.^7^;

c Anderson et al.^8^;

d Sigma-Aldrich catalogue;

e Rosini.^53^;

f Elsenbaumer and Mosher^25^;

g Expected sign, numerical value not reported in the literature.

h Numerical value different from zero due to slightly asymmetric optimized geometry.

## Conclusions

In conclusion, in this work, we have presented the interpretation of VCD spectra in the CD stretching region of three prototypical deuterated molecules whose spectra had been measured in the past but had not been fully elucidated until now. This first systematic investigation of inherently symmetric HC*D vibrational chromophores highlights the importance of anharmonicity, as well as the capabilities of a relatively cheap method like VPT2 to capture even minute details of the VCD band shapes. The very good match between experimental and computed data is very encouraging for tackling other cases^56–61^ where more than one CD bond is present and excitonic contributions might be important,^62–64^ as can happen with inherently dissymmetric chromophores.^32^ For the definition of inherently symmetric and inherently dissymmetric chromophores, the interested reader might refer to the paper by Moscowitz.^31^

Here, the significant impact of anharmonicity raises doubts about the straightforward use of the bands in the CD stretching region to directly infer the configuration of the HC*D group. This is because the 1800–2300 region is densely populated with two-quanta overtone and combination transitions, which significantly modulate the signal compared to the pure fundamental transition assumed to be isolated (see, for instance, the spectra reported in Stephens and Clark^21^ as well as our previous works^20,24^ ). Yet, the reliability of full anharmonic treatment makes the use of this region useful for configuration assignment.

Finally, vibrational corrections in the anharmonic framework appear to be of utmost importance also for the correct prediction of optical rotation data for molecules containing the HC*D group.

## Supplementary Material



## Notes

Additional simulated data, regarding VCD regions of lesser importance and description of anharmonic interaction terms (Section S1); a brief historical description for the zero-point energy vibrational correction terms (Section S2); the current approach to vibrational corrections to optical rotation, together with the complete results for the molecules treated here (Section S3); the Cartesian coordinates of the molecules dealt with here (Section S4) (PDF)
