# Supplementary material for: The Role of Anharmonicity in the HC*D Chromophore in Vibrational Circular Dichroism Spectra and Optical Rotation Data
Source: J Phys Chem A. 2025 Jul 14;129(29):6615–22. doi: 10.1021/acs.jpca.5c03064 (PMC12302218; doi:10.1021/acs.jpca.5c03064)
Supplement: Supplementary file 1 [file jp5c03064_si_001.pdf]

Supporting Information: The role of Anharmonicity in the HC*D Chromophore in Vibrational Circular Dichroism Spectra and Optical Rotation data

Marco Fusè,[*,†] Giovanna Longhi,[†,¶] Giuseppe Mazzeo,[†] Julien Bloino,[‡] and Sergio Abbate[*,†,¶]

†*Dipartimento di Medicina Molecolare e Traslazionale, Università di Brescia, Viale Europa 11, 25123 Brescia, Italy*

‡*Scuola Normale Superiore, Piazza dei Cavalieri, 56125 Pisa, Italy*

¶*Istituto Nazionale di Ottica (INO), CNR, Research Unit of Brescia, c/o CSMT, VIA Branze 45, 25123 Brescia, Italy*

E-mail: marco.fuse@unibs.it; sergio.abbate@unibs.it

S1 Additional Simulated Data

Table S1: Harmonic (ω) and anharmonic (ν) DVPT2 energies (cm^{-1}) of (R)-(−)-neopentyl-1d-chloride and (R)-(−)-neopentyl-1d-bromide. Deperturbed dipole strength (DS) ($\times 10^{-40}$) and rotatory strength (RS) ($\times 10^{-44}$) in cgs units.

(R)-(−)-neopentyl-1d-chloride						
Transition	ω	ν	Harm. DS	Anh. DS	Harm. RS	Anh. RS
1_{35}	2277.473	2198.589	15.27	17.07	0.338	0.382
$1_{21}1_{20}$	2265.290	2213.305		0.01		-0.034
$1_{25}1_{15}$	2264.474	2208.434		0.04		-0.031
$1_{25}1_{17}$	2288.432	2233.425		0.01		0.006
**(R)-(−)-neopentyl-1d-bromide**						
Transition	ω	ν	Harm. DS	Anh. DS	Harm. RS	Anh. RS
1_{35}	2289.175	2212.752	9.59	10.88	1.119	0.950
$1_{21}1_{20}$	2244.532	2195.340		0.01		-0.015
$1_{25}1_{15}$	2252.987	2195.918		0.05		-0.028
$1_{25}1_{17}$	2284.031	2229.108		0.01		0.001

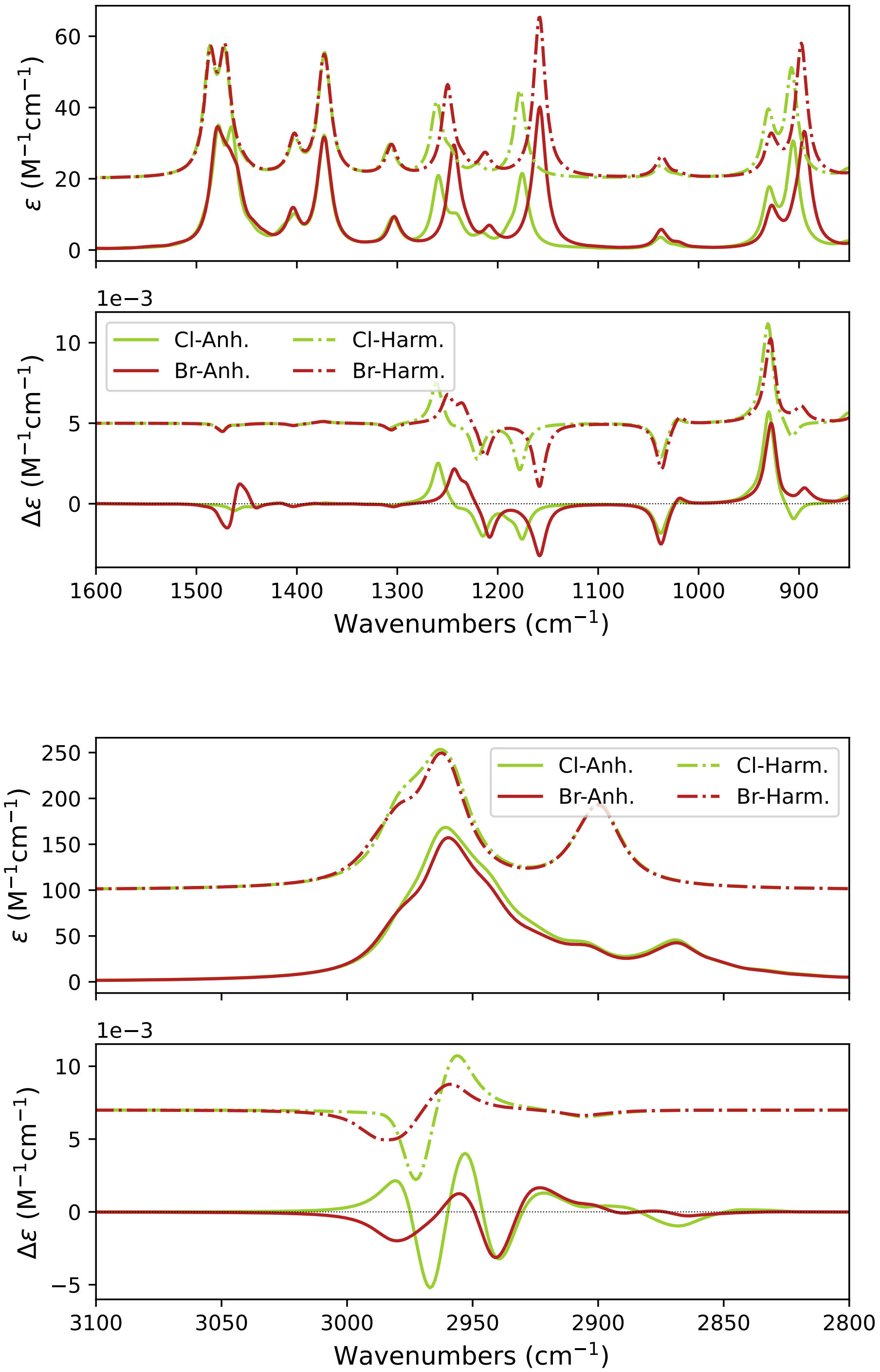

Figure S1: Calculated harmonic (dashed lines) and anharmonic (solid lines) IR and VCD spectra of (*R*)-(−)-neopentyl-1d-chloride and (*R*)-(−)-neopentyl-1d-bromide in the fingerprint (upper panels) and CH-stretching (lower panels) regions. In the two regions, harmonic frequencies were scaled by 0.978 and 0.958, respectively.

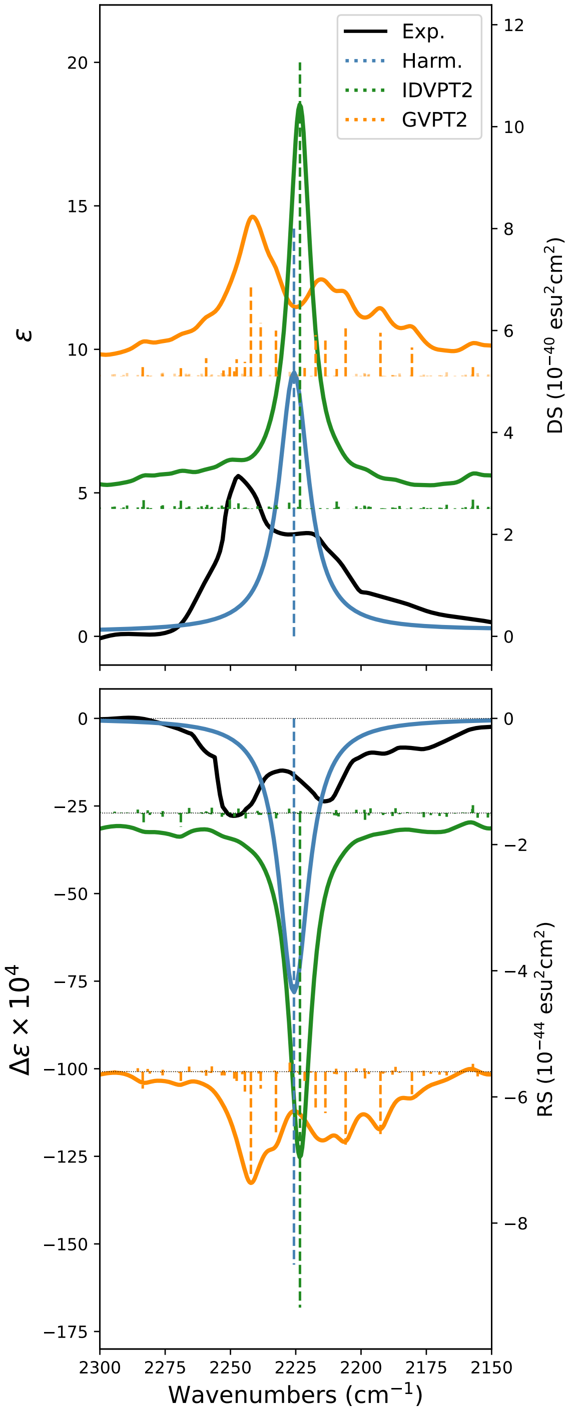

Figure S2: Comparison of experimental and calculated IR (top) and VCD (bottom) spectra of (R)-(+)-1-exo-d$_1$-camphor in the CD-stretching region. Experimental spectra are in black, calculated calculated harmonic spectra are in blue, deperturbed spectra before variational correction (IDVPT2) are in green and calculated GVPT2 spectra are in orange. A scaling factor of 0.977 was applied to harmonic frequencies. The dipole strengths (DS) and rotatory strengths (RS) of the transitions are also reported.

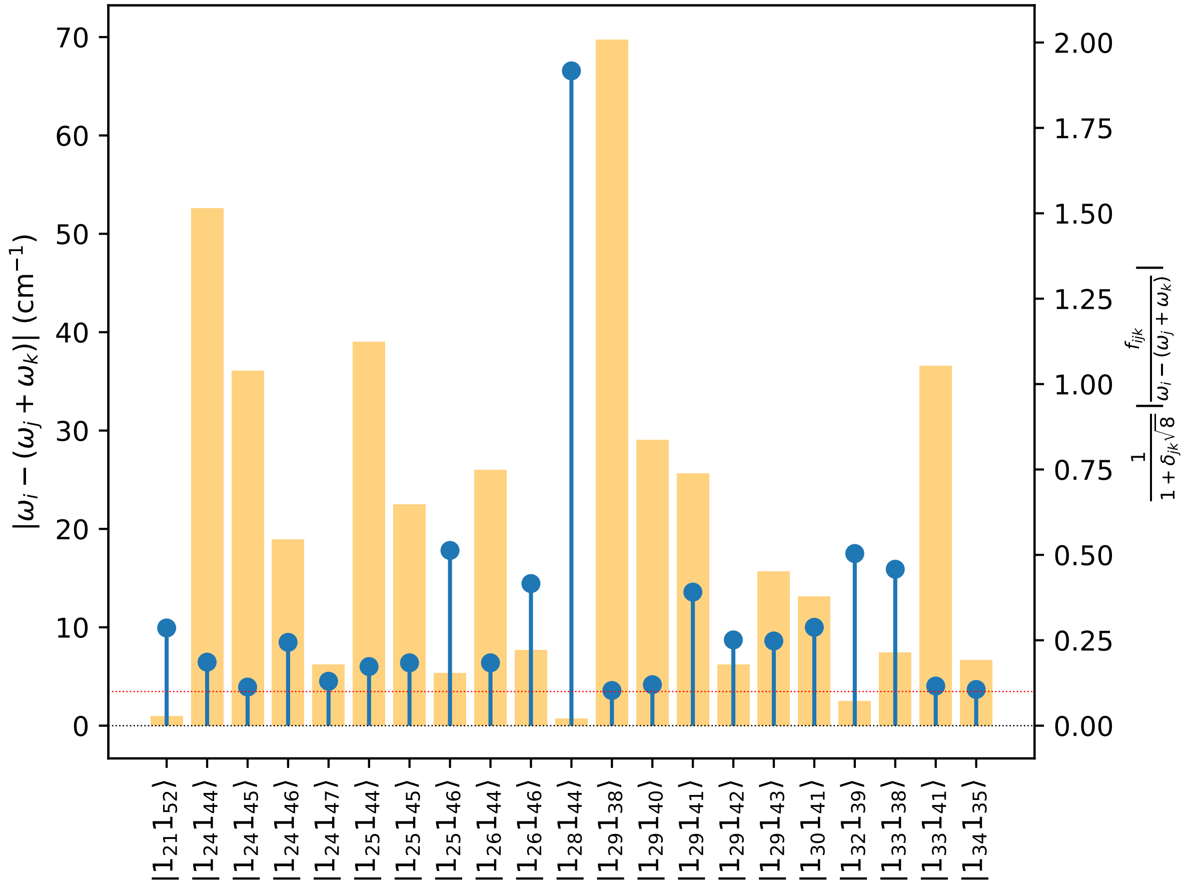

Figure S3: (R)-(+)-1-exo-d$_1$-camphor: active Fermi resonances between state $|1_{60}\rangle$ and the listed two quanta transitions. The harmonic energy difference between the fundamental and combination bands is reported as orange lines, while blue pins correspond to the coefficient of the first-order perturbation to the vibrational wave function, used as metrics to identify the magnitude of Fermi resonances, with particular emphasis on intensities, more sensitive.[S1] The horizontal red line represents the default threshold value employed in the test.

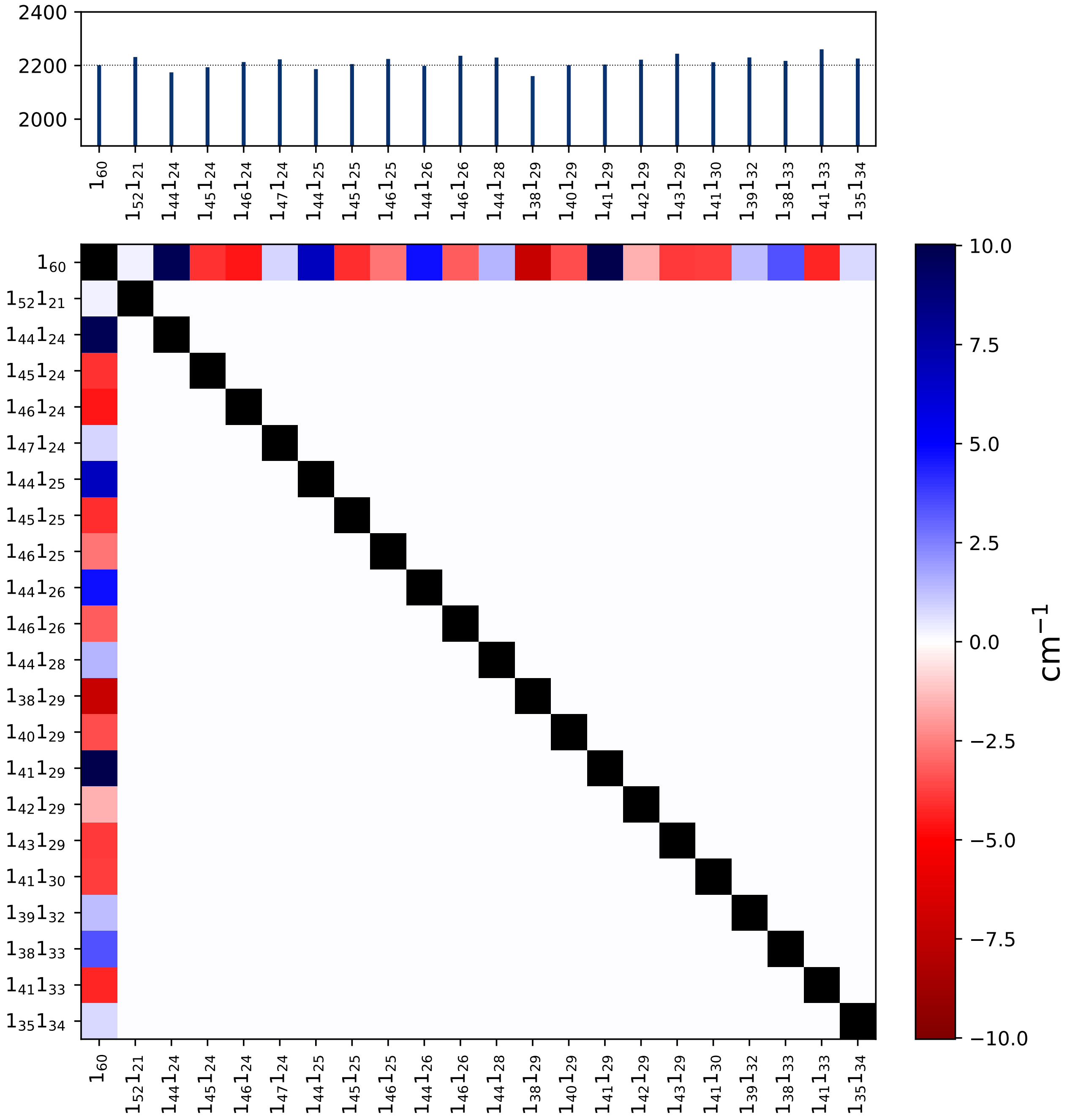

Figure S4: (R)-(+)-1-exo-d$_1$-camphor: graphical representation of the variational polyad involving the CD stretching fundamental (1_{60}). The magnitude of the diagonal elements is reported in the top panel, while the off-diagonal ones are reported in the bottom panel.

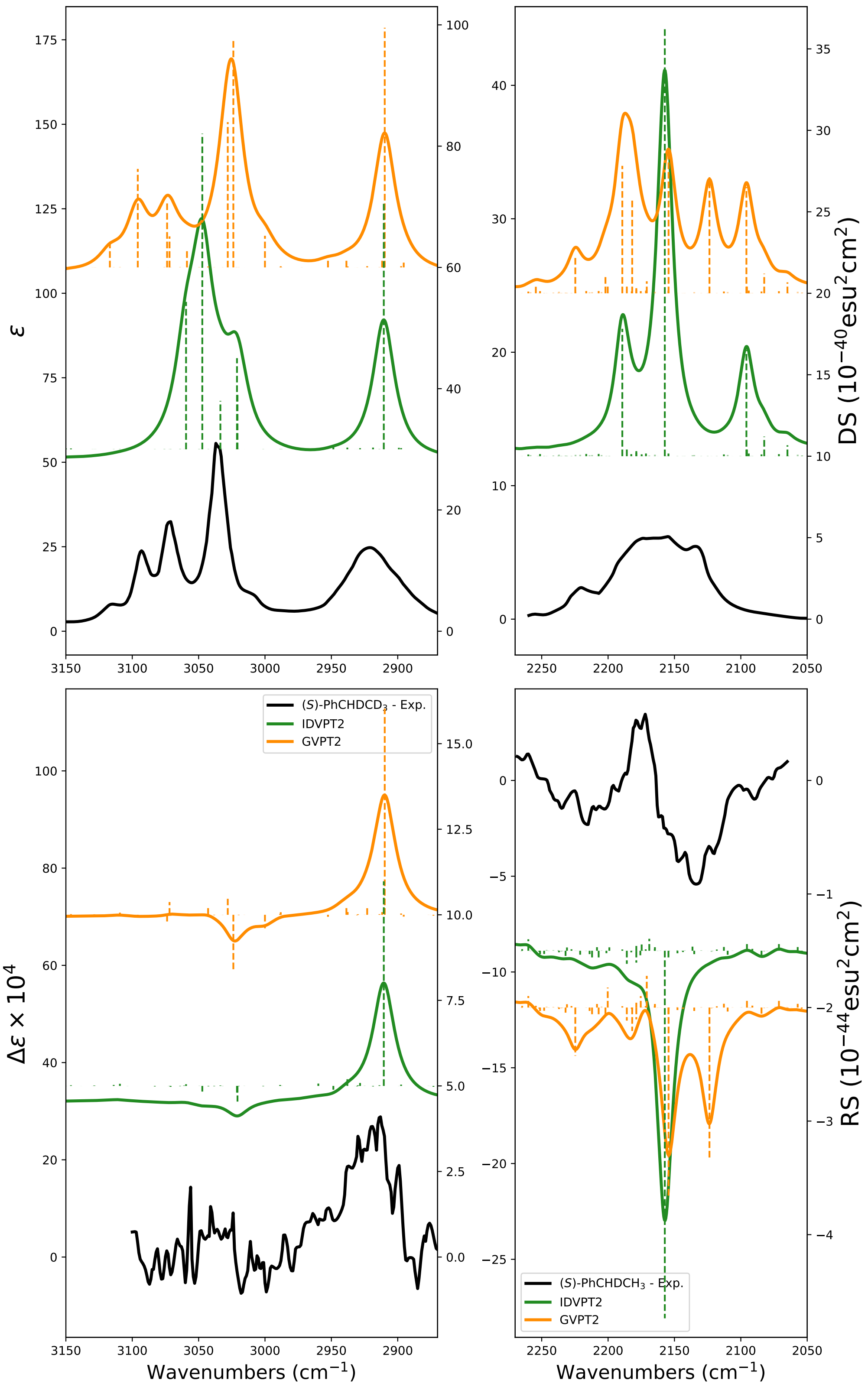

Figure S5: Comparison of experimental and calculated IR and VCD spectra of (*S*)-(+)-1-phenylethane-1,2,2,2-d$_4$ in the CH-stretching region (left) and of (*S*)-(+)-1-phenylethane-1-d$_1$ in the CD-stretching region (right). Experimental spectra are in black, calculated anharmonic IDVPT2 and GVPT2 spectra are in green and orange respectively.

Table S2: Anharmonic (ν) GVPT2 energies (cm^{-1}), rotational strengths (cgs), variational states and variational coefficients of the deperturbed state of selected transitions of (R)-(+)-1-exo-d_1-camphor. The labels are the one used in figure S6.

Label	ν (cm^{-1})	RS $\times 10^{44}$ (cgs)	var. state	var. coefficients deperturbed states
1	2180.5	-0.38	1209	+0.955554 $\|1_{38}1_{29}\rangle$<br>+0.235787 $\|1_{60}\rangle$<br>-0.146650 $\|1_{44}1_{24}\rangle$
2	2192.6	-1.05	1225	+0.882642 $\|1_{44}1_{24}\rangle$<br>-0.340566 $\|1_{60}\rangle$<br>+0.241677 $\|1_{38}1_{29}\rangle$<br>+0.145303 $\|1_{44}1_{25}\rangle$<br>+0.103699 $\|1_{41}1_{29}\rangle$
3	2205.9	-1.16	1236	+0.831742 $\|1_{44}1_{25}\rangle$<br>-0.339953 $\|1_{44}1_{24}\rangle$<br>-0.328081 $\|1_{60}\rangle$<br>+0.166662 $\|1_{41}1_{29}\rangle$<br>-0.138538 $\|1_{45}1_{24}\rangle$<br>+0.106883 $\|1_{44}1_{26}\rangle$<br>+0.101264 $\|1_{38}1_{29}\rangle$
4	2213.7	-0.66	1242	+0.695354 $\|1_{45}1_{24}\rangle$<br>+0.447035 $\|1_{44}1_{25}\rangle$<br>+0.330920 $\|1_{60}\rangle$<br>-0.275142 $\|1_{41}1_{29}\rangle$<br>-0.226439 $\|1_{44}1_{26}\rangle$<br>+0.188942 $\|1_{44}1_{24}\rangle$<br>+0.122083 $\|1_{40}1_{29}\rangle$
5	2217.4	-0.62	1243	+0.682275 $\|1_{45}1_{24}\rangle$<br>+0.428956 $\|1_{44}1_{26}\rangle$<br>+0.353968 $\|1_{41}1_{29}\rangle$<br>-0.295504 $\|1_{60}\rangle$<br>-0.229901 $\|1_{44}1_{25}\rangle$<br>-0.178664 $\|1_{40}1_{29}\rangle$<br>-0.138787 $\|1_{44}1_{24}\rangle$<br>-0.121580 $\|1_{45}1_{25}\rangle$
6	2232.6	-0.96	1256	+0.573189 $\|1_{41}1_{30}\rangle$<br>+0.506394 $\|1_{46}1_{24}\rangle$<br>+0.440754 $\|1_{41}1_{29}\rangle$<br>+0.293020 $\|1_{60}\rangle$<br>-0.241864 $\|1_{45}1_{25}\rangle$<br>-0.143681 $\|1_{38}1_{33}\rangle$<br>+0.119106 $\|1_{44}1_{26}\rangle$<br>-0.110435 $\|1_{40}1_{29}\rangle$
7	2242.2	-1.67	1264	+0.586012 $\|1_{38}1_{33}\rangle$<br>+0.430834 $\|1_{60}\rangle$<br>+0.341336 $\|1_{42}1_{29}\rangle$<br>-0.290810 $\|1_{46}1_{24}\rangle$<br>+0.267213 $\|1_{41}1_{29}\rangle$<br>+0.250569 $\|1_{46}1_{25}\rangle$<br>-0.215604 $\|1_{41}1_{30}\rangle$<br>-0.122653 $\|1_{45}1_{25}\rangle$<br>-0.109167 $\|1_{47}1_{24}\rangle$
8	2244.5	-0.31	1266	+0.923922 $\|1_{42}1_{29}\rangle$<br>-0.198523 $\|1_{46}1_{25}\rangle$<br>-0.176920 $\|1_{60}\rangle$<br>+0.155863 $\|1_{47}1_{24}\rangle$<br>-0.126525 $\|1_{38}1_{33}\rangle$

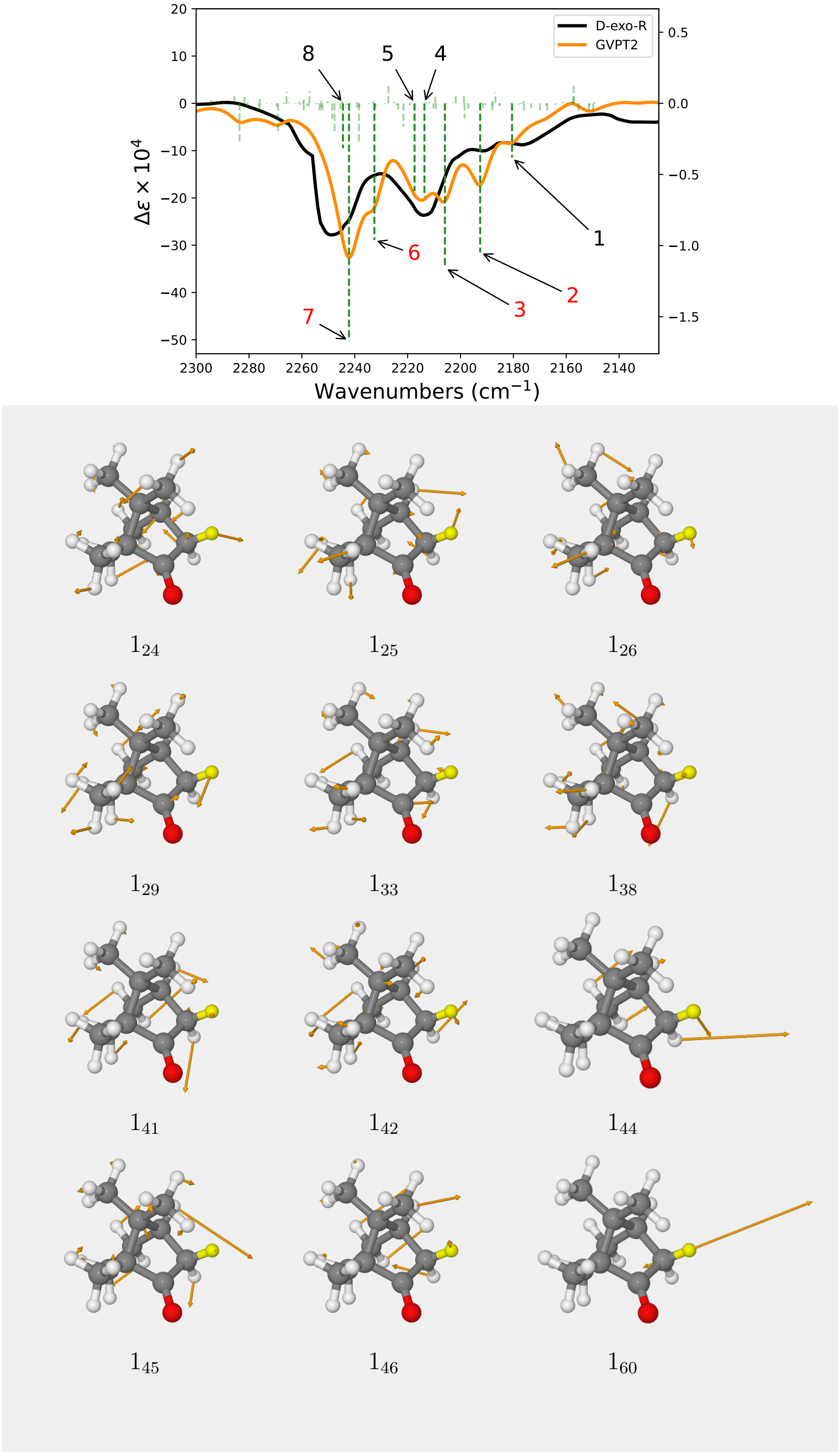

Figure S6: Top panel: comparison of experimental (black) and simulated VCD spectra of (R)-(+)-1-exo-d_1-camphor. The most intense transition were labeled and assignment is give in table S2. Bottom panel: graphical representation of the normal modes involved in the states contributing to the most intense VCD features.

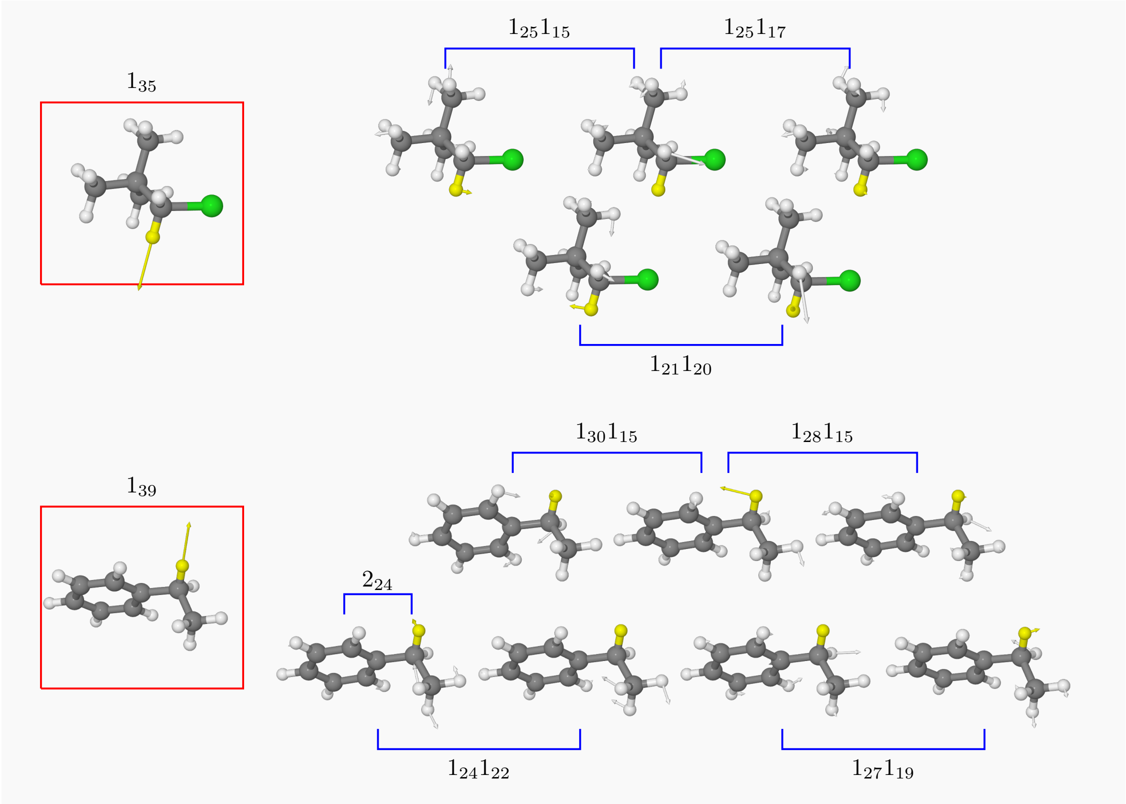

Figure S7: Graphical representation of the normal modes involved in the states contributing to the most intense VCD/IR features. On the left, in a red square, the fundamentals in the region of interest, on the right, the overtones and combination bands involved.
Top: The three most intense VCD/IR features of (R)-(−)-neopentyl-1d-chloride showed in Fig. 1 in the main text are related to the CD-stretching fundamental 1_{35} and three combination states, $1_{25}1_{15}$, $1_{25}1_{17}$ and $1_{21}1_{20}$; bottom: the four most intense VCD/IR features of (S)-(+)-1-phenylethane-1-d$_1$ in Fig. 3 in the main text are caused by interactions between the fundamental of the CD-stretching mode 1_{39} and five overtone/combination modes, 2_{24}, $1_{24}1_{22}$, $1_{27}1_{19}$, $1_{28}1_{15}$, $1_{30}1_{15}$. In both cases, the main numbers denote the number of excitation quanta and the subscripts the normal-mode indexes.

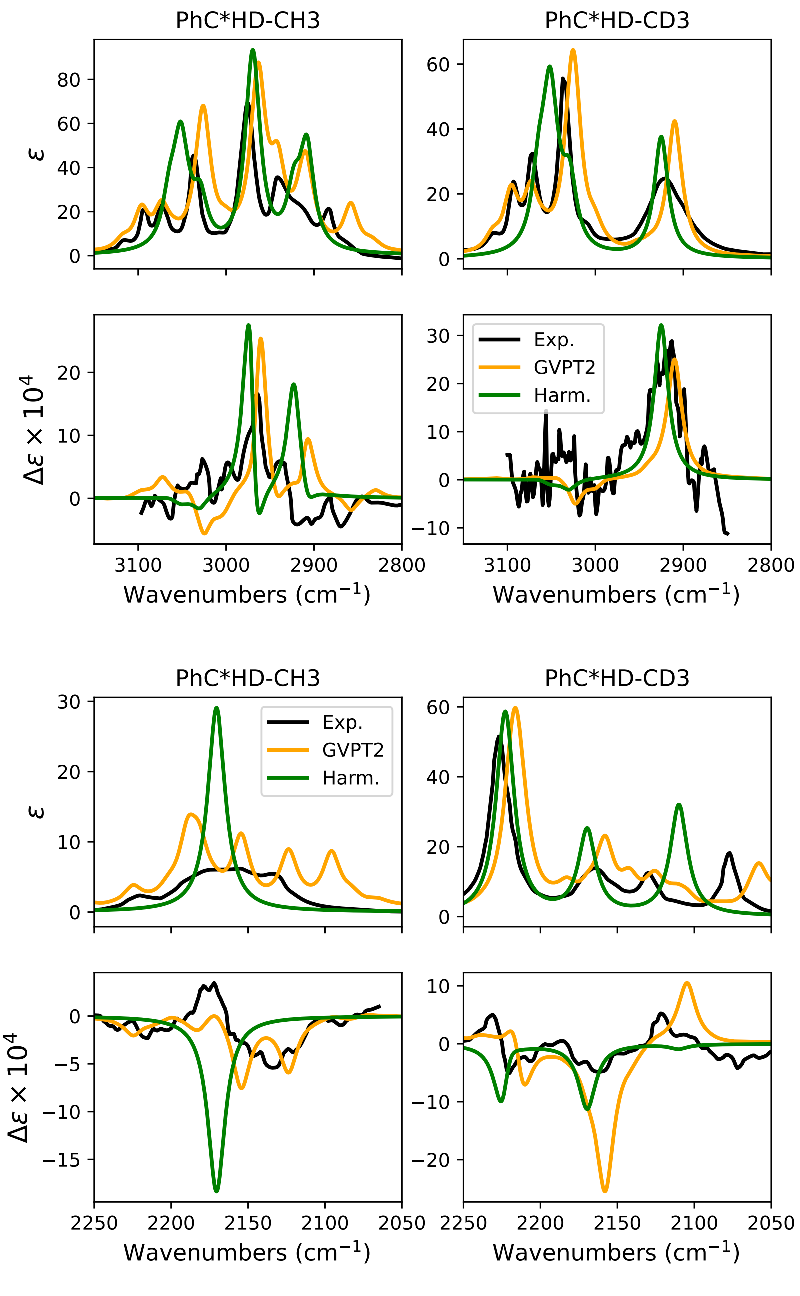

Figure S8: Comparison of experimental and calculated IR and VCD spectra of (S)-(+)-1-phenylethane-1,2,2,2-d_4 and (S)-(+)-1-phenylethane-1-d_1 in the CH-stretching region (top) and of in the CD-stretching region (bottom). The calculated harmonic frequencies were scaled by 0.96 and 0.97 factors respectively.

S2 Historical Note on Vibrational Contributions to Optical Rotation

The equation proposed by Kirkwood to compute the optical rotation (see Eq. 35 or Ref. S2 was derived under the assumption of N polarizable groups interacting through electric dipole-electric dipole interaction-type forces. The mean polarizability and relative polarizability anisotropy are specified but the magnetic dipole moment terms are neglected in the model.

$$g^{(0)} = -\frac{1}{6}\sum_{i>k=1}^{N} \alpha_i \alpha_k \beta_i \beta_k G_{ik} \mathbf{R}_{ik} \cdot (\mathbf{b}_i \times \mathbf{b}_k); \tag{1}$$

where:

$$G_{ik} = \mathbf{b}_i \cdot \mathbf{T}_{ik} \cdot \mathbf{b}_k = \frac{1}{R_{ik}^3}\left[\mathbf{b}_i \cdot \mathbf{b}_k - 3\frac{(\mathbf{b}_i \cdot \mathbf{R_{ik}})(\mathbf{b}_k \cdot \mathbf{R_{ik}})}{R_{ik}^2}\right]$$

and $\mathbf{R}_{ik} = \mathbf{R}_k - \mathbf{R}_i$ and $\mathbf{b}_1^{(k)}$, $\mathbf{b}_2^{(k)}$, $\mathbf{b}_3^{(k)}$ are unit vectors defining the optical axes for group k.

The other quantities are defined as,

$$\alpha_i = \frac{1}{3}(\alpha_{11}^1 + \alpha_{22}^i + \alpha_{33}^i) \qquad \beta_i = \frac{\alpha_{11}^1 - \alpha_{22}^i}{\alpha_i} = 3\frac{\alpha_{11}^1 - \alpha_{22}^i}{\alpha_{11}^1 + 2\alpha_{22}^i} \tag{2}$$

and represent the mean polarizability and relative polarizability anisotropy in the case of cylindrical symmetry (the "(1)" axis is different from axes "(2)" and "(3)", which are equivalent).

Expanding Kirkwood expression for $g^{(0)}$ in terms of the normal vibrational coordinates, Fickett obtained the averaged optical rotation $\langle g^{(0)} \rangle$:[S3]

$$\begin{aligned}\langle g^{(0)} \rangle = \frac{1}{6}\sum_{i\neq k=1}^{N} \alpha_i\beta_i \Bigg[\left(\sum_l \alpha_l^{(k)\prime} \beta_l^{(k)\prime} \langle q_l \rangle\right) (G_{ik} + G'_{ik})(\mathbf{R}_{ik} \cdot (\mathbf{b}_i \times \mathbf{b}_k)) + \\ \alpha_i\beta_i \left(G'_{ik}\mathbf{R}_{ik} + G_{ik}\left(\sum_l \left(\frac{\partial \mathbf{R}_{ik}}{\partial q_l}\langle q_l \rangle\right)\right)\right) \cdot (\mathbf{b}_i \times \mathbf{b}_k)\Bigg]\end{aligned} \tag{3}$$

where:

$$\alpha_l^{(k)'} = \frac{1}{3}\left\{\left(\frac{\partial \alpha_{11}^{(k)}}{\partial q_l}\right)_0 + 2\left(\frac{\partial \alpha_{22}^{(k)}}{\partial q_l}\right)_0\right\}$$

$$\alpha_l^{(k)'}\beta_l^{(k)'} = \left(\frac{\partial \alpha_{11}^{(k)}}{\partial q_l}\right)_0 - \left(\frac{\partial \alpha_{22}^{(k)}}{\partial q_l}\right)_0$$

and

$$G'_{ik} = \mathbf{b}_i\left\{\sum_l \left(\frac{\partial \mathbf{T}_{ik}}{\partial q_l}_0 \langle q_l\rangle\right)\right\}\mathbf{b}_k$$

For the R_1R_2CHD case, it is possible to further simplify the equation to

$$\langle g^{(0)}\rangle = \frac{1}{6}\sum_{i\neq H,D}\alpha_i\beta_i\Big[\alpha^{(H)'}\beta^{(H)'}\left(G_{iH}+G'_{iH}\right)\left(\mathbf{R}_{iH}\cdot(\mathbf{b}_i\times\mathbf{b}_H)\right)\langle x_H\rangle + \alpha^{(D)'}\beta^{(D)'}\left(G_{iD}+G'_{iD}\right)\left(\mathbf{R}_{iD}\cdot(\mathbf{b}_i\times\mathbf{b}_D)\right)\langle x_D\rangle\Big] \quad (4)$$

Here x_H and x_D represents the elongations of the CH or CD bond from equilibrium distance and the sum extends over all groups other than H and D.

Table S3: Experimental Mean Polarizability values ($\alpha = [1/3(\alpha_{xx}+\alpha_{yy}+\alpha_{zz}])(\mathring{A}^3)$ and few ancillary related quantities for molecular models useful for the present investigation. Data taken from: (a) Le Bel;[S4] (b) Sherman;[S5] (c) Hahn and Kerl[S6] ; (d) Gough;[S7] (e) Gussoni, Rui and Zerbi[S8]

	Bell[a]	Sherman[b]	Hahn/Kerl[c]	Gough[d]	Gussoni/Zerbi[e]
H_2/D_2	0.779 (i)				
HCL/DCl	2.49 (ii)				
HBr/DBr	3.39 (iii)				
CH_4/CD_4	2.47 (iv)		2.85-2.93		
Neopent.			9.70-10.15	8.871	
Benzene		10.4 (v)			9.96
Percent difference of deuterated to undeuterated species mean polarizability					
$\delta = 100[(\alpha_H \alpha_D)/\alpha_H]$					
(i) 1.315	(ii) 0.222	(iii) 0.144	(iv) 1.45		
Dimensionless anisotropy values (v)					
(definition for cylindrical symmetry benzene)					
$\beta = 3\frac{\gamma}{\bar{\alpha}} = \frac{(\alpha_{xx}-\alpha_{zz})}{(\alpha_{xx}+\alpha_{yy}+\alpha_{zz})} = \frac{(\alpha_{yy}-\alpha_{zz})}{(\alpha_{xx}+\alpha_{yy}+\alpha_{zz})} = 1.07$					

S3 Vibrational Averaged Optical Rotation

The optical rotation can be obtained as the trace of the electric dipole-magnetic dipole polarizability tensor. It is a combination of the equilibrium property and the effects of the nuclear motions. Thus, it can be written in two separate terms where the equilibrium term can be obtained from linear response theory and the vibrational contribution from perturbation theory as zero-point correction.[S9–S12]

$$[\alpha]^{\mathrm{zpvc}} = [\alpha]^{\mathrm{eq}} + [\alpha]^{\mathrm{vib}} \tag{5}$$

$$\begin{aligned}[\alpha]^{\mathrm{vib}} &= [\alpha]^{\mathrm{mec}} + [\alpha]^{\mathrm{ele}} \\ &= -\frac{1}{4}\sum_{i=1}^{N}\frac{1}{\omega_i^2\sqrt{\mu_i}}\left(\frac{\partial[\alpha]}{\partial Q_i}\right)_0\sum_{j=1}^{N}\frac{\mathsf{f}_{ijj}}{\omega_j\mu_j\sqrt{\mu_i}} + \frac{1}{4}\sum_{i=1}^{N}\frac{1}{\omega_i\mu_i}\left(\frac{\partial^2[\alpha]}{\partial Q_i^2}\right)_0\end{aligned} \tag{6}$$

Here, the first term $[\alpha]^{1\mathrm{st}}$ depends on the anharmonicity of the potential energy surface, and is thus connected to the mechanical anharmonicity. The second term $[\alpha]^{2\mathrm{nd}}$, instead, accounts for the property surface corrections, which is often referred to as electrical anharmonicity.

Temperature effects can also be accounted for, thus leading to the values reported in Table 1 in the main text.[S11,S13]

$$\begin{aligned}[\alpha]^{T} = [\alpha]^{\mathrm{eq}} &- \frac{1}{4}\sum_{i=1}^{N}\frac{1}{\omega_i^2\sqrt{\mu_i}}\left(\frac{\partial[\alpha]}{\partial Q_i}\right)_0\sum_{j=1}^{N}\frac{\mathsf{f}_{ijj}}{\omega_j\mu_j\sqrt{\mu_i}}\coth\left(\frac{\epsilon_j}{2k_BT}\right) \\ &+ \frac{1}{4}\sum_{i=1}^{N}\frac{1}{\omega_i\mu_i}\left(\frac{\partial^2[\alpha]}{\partial Q_i^2}\right)_0\coth\left(\frac{\epsilon_i}{2k_BT}\right)\end{aligned} \tag{7}$$

where ϵ_i is the fundamental energy associated to mode i and μ its reduced mass.

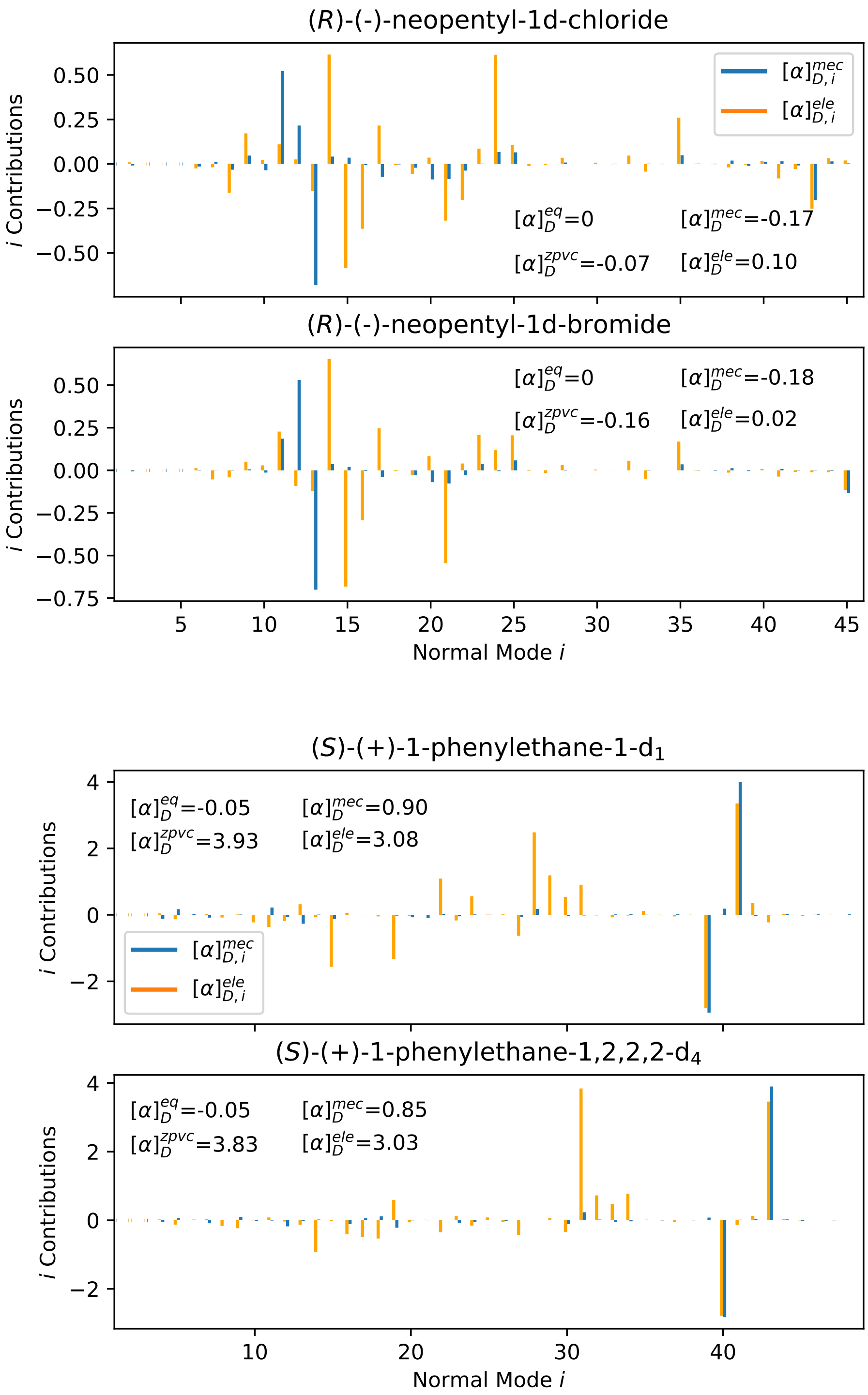

Figure S9: Graphical representation of the $[\alpha]^{\mathrm{mec}}$ and $[\alpha]^{\mathrm{ele}}$ contribution to the zero-point vibrational correction to $[\alpha]$.

S4 Cartesian Coordinates

neopentyl-chloride

```
17
B3LYP/Def2-TZVP
C           0.456382     0.888543    -0.000420
H           0.476307     1.520068     0.885719
H           0.476297     1.519278    -0.887122
C          -0.781481    -0.017270     0.000003
C          -1.997901     0.927644    -0.000105
H          -2.006248     1.568847    -0.884700
H          -2.924108     0.349738     0.000248
H          -2.005948     1.569441     0.884063
C          -0.816462    -0.893354     1.258985
H          -1.726340    -1.496778     1.276041
H           0.038243    -1.568999     1.295465
H          -0.802735    -0.282965     2.165378
C          -0.816827    -0.894101    -1.258450
H           0.037814    -1.569835    -1.294735
H          -1.726760    -1.497458    -1.274925
H          -0.803270    -0.284253    -2.165210
Cl          2.041440     0.006126    -0.000017
```

neopentyl-bromide

```
17
B3LYP/Def2-TZVP
C          -0.117821     0.910010    -0.000118
H          -0.095475     1.537204     0.887466
H          -0.095478     1.536989    -0.887853
C          -1.336434    -0.018092     0.000001
C          -2.565917     0.913477    -0.000013
H          -2.582019     1.554373    -0.884535
H          -3.483968     0.322521     0.000082
H          -2.581930     1.554516     0.884407
C          -1.365969    -0.894126     1.258952
H          -2.273622    -1.500856     1.276730
H          -0.508795    -1.566807     1.294346
H          -1.353293    -0.283871     2.165395
C          -1.366086    -0.894313    -1.258818
H          -0.508927    -1.567013    -1.294184
H          -2.273751    -1.501029    -1.276431
H          -1.353475    -0.284193    -2.165354
Br          1.646403     0.002755    -0.000003
```

Camphor

```
27
B2PLYP/def2-TZVP
C           0.054339     0.594823    -1.631889
C           0.291422    -0.499919    -0.549801
C          -0.605516     1.256658     0.596271
C          -0.599567     1.770626    -0.855735
H           0.987337     0.875294    -2.122349
H          -0.605058     0.201570    -2.406170
H          -0.032177     2.696893    -0.947938
H          -1.606924     1.975583    -1.215459
C          -0.908401    -0.260704     0.421861
C           0.856876     1.255385     1.065173
H           0.973126     1.069200     2.133240
H           1.397025     2.174771     0.834683
H          -1.289851     1.786093     1.259737
C          -0.805045    -1.067859     1.719814
H          -1.010299    -2.122245     1.528048
H           0.175416    -1.012053     2.191501
H          -1.544198    -0.718071     2.443864
C          -2.278588    -0.544964    -0.193538
```

H	-2.408943	-1.615935	-0.358538
H	-3.067094	-0.222744	0.490714
H	-2.442300	-0.042481	-1.144250
C	1.437562	0.077541	0.278091
O	2.582275	-0.309606	0.302022
C	0.548327	-1.887742	-1.087161
H	1.451630	-1.895850	-1.698126
H	0.699780	-2.605376	-0.279957
H	-0.284114	-2.230876	-1.703691

Phenylethane

18
B3LYP/Def2-TZVP

C	1.632330	-1.200610	0.095596
C	0.270506	-1.197475	-0.183143
C	-0.432768	-0.000087	-0.324936
C	0.270382	1.197395	-0.183285
C	1.632202	1.200705	0.095452
C	2.318562	0.000091	0.237284
H	2.158829	-2.141614	0.197553
H	-0.254354	-2.139420	-0.297672
H	-0.254577	2.139271	-0.297929
H	2.158606	2.141775	0.197296
H	3.379943	0.000161	0.450987
C	-1.919115	-0.000175	-0.589657
H	-2.180488	0.875899	-1.189033
H	-2.180459	-0.876553	-1.188597
C	-2.758071	0.000131	0.695599
H	-2.543180	0.881586	1.302820
H	-3.825320	0.000062	0.463864
H	-2.543157	-0.881021	1.303251

References

(S1) Yang, Q.; Bloino, J. An Effective and Automated Processing of Resonances in Vibrational Perturbation Theory Applied to Spectroscopy. *The Journal of Physical Chemistry A* **2022**, *126*, 9276–9302.

(S2) Kirkwood, J. G. On the Theory of Optical Rotatory Power. *The Journal of Chemical Physics* **1937**, *5*, 479–491.

(S3) Fickett, W. Zero-point Vibrational Contributions to the Optical Rotatory Power of Isotopically Dissymmetric Molcules. *Journal of the American Chemical Society* **1952**, *74*, 4204–4205, Publisher: American Chemical Society.

(S4) Bell, R. P. Polarisibility and internuclear distance. *Trans. Faraday Soc.* **1942**, *38*, 422–429.

(S5) Jeffrey Sherman, B.; Sen, S.; Galiatsatos, V. Quantitative prediction of molecular optical polarizability anisotropy: benzene, substituted benzene and rigid-rod oligomers. *Polymer* **1996**, *37*, 1759–1764.

(S6) Hohm, U.; Kerl, K. Anomalous Behaviour of the Mean Dipole Polarizability α of Neopentane C(CH3)4 in the Temperature Range between 250 K and 360 K. *Zeitschrift für Naturforschung A* **1991**, *46*, 983–988.

(S7) Gough, K. M. Theoretical analysis of molecular polarizabilities and polarizability derivatives in hydrocarbons. *The Journal of Chemical Physics* **1989**, *91*, 2424–2432.

(S8) Gussoni, M.; Rui, M.; Zerbi, G. Electronic and relaxation contribution to linear molecular polarizability. An analysis of the experimental values. *Journal of Molecular Structure* **1998**, *447*, 163–215.

(S9) Ruud, K.; Taylor, P. R.; Åstrand, P.-O. Zero-point vibrational effects on optical rotation. *Chemical Physics Letters* **2001**, *337*, 217–223.

(S10) Mort, B. C.; Autschbach, J. Magnitude of Zero-Point Vibrational Corrections to Optical Rotation in Rigid Organic Molecules: A Time-Dependent Density Functional Study. *The Journal of Physical Chemistry A* **2005**, *109*, 8617–8623.

(S11) Egidi, F.; Barone, V.; Bloino, J.; Cappelli, C. Toward an Accurate Modeling of Optical Rotation for Solvated Systems: Anharmonic Vibrational Contributions Coupled to the Polarizable Continuum Model. *Journal of Chemical Theory and Computation* **2012**, *8*, 585–597, Publisher: American Chemical Society.

(S12) Faintich, B.; Parsons, T.; Balduf, T.; Caricato, M. Theoretical Study of the Isotope Effect in Optical Rotation. *The Journal of Physical Chemistry A* **2024**, *128*, 8045–8059, Publisher: American Chemical Society.

(S13) Mort, B. C.; Autschbach, J. A Pragmatic Recipe for the Treatment of Hindered Rotations in the Vibrational Averaging of Molecular Properties. *ChemPhysChem* **2008**, *9*, 159–170.
